# CD47-blocking therapies stimulate macrophage cytokine secretion and are effective in a model of peritoneal carcinomatosis

**DOI:** 10.1186/2051-1426-3-S2-P248

**Published:** 2015-11-04

**Authors:** Kipp Weiskopf, Aaron Ring, K Christopher Garcia, Irving Weissman

**Affiliations:** 1 Stanford University School of Medicine, Stanford, CA, USA

## 

Peritoneal carcinomatosis is an advanced state of malignancy often arising from gastrointestinal or ovarian cancers. It is characterized by dissemination of tumors throughout the peritoneal cavity and is associated with 5-year survival rates less than 25%. Since the peritoneal cavity is highly enriched with macrophages, we investigated the efficacy of CD47-blocking therapies for peritoneal carcinomatosis. CD47 is a molecule that is highly expressed on the surface of many tumors, where it acts as a myeloid-specific immune checkpoint. By binding to SIRPa, an inhibitory receptor on macrophages and other myeloid cells, CD47 is able to transduce inhibitory signals that prevent macrophage phagocytosis.

We first we assessed the potency of various CD47-blocking reagents at stimulating phagocytosis in vitro. Using human macrophages and human colon cancer cells, we found that CD47-blocking reagents induced phagocytosis in a dose-dependent manner. The high-affinity SIRPa-Fc fusion proteins[1] induced phagocytosis with greater potency compared to anti-CD47 antibodies (EC_50_ 0.8351 vs. 4.669 nM, *p* = 0.0003) or other CD47-blocking reagents. Using multiplex profiling of 51 cytokines, we identified a unique cytokine signature that correlated with macrophage phagocytosis. We next established a xenograft model of peritoneal carcinomatosis in which mice progressed to form malignant ascites. When tumor-bearing mice were treated with CD47-blocking therapies, we detected a cytokine signature within the tumor microenvironment. In particular, MCP-3 was elevated within both the ascites fluid and serum of tumor-bearing mice after treatment with high-affinity SIRPa-Fc fusion proteins (*p* < 0.0001) and may serve as a clinical biomarker of response to therapy. Last, we investigated the therapeutic efficacy of CD47-blocking therapies for peritoneal carcinomatosis. Animals treated with high-affinity SIRPa-Fc fusion proteins exhibited inhibition of tumor growth (*p* < 0.05) and prolonged survival (*p* = 0.0022) relative to control animals. Since the high-affinity SIRPa-Fc fusion proteins bind both human and mouse CD47, this model provides insight into therapeutic efficacy in the setting of an ‘antigen sink’ and toxicity. The main toxicity observed was a reduction in hematocrit (41.78% vs. 35.63%, *p* < 0.001). CD47-blocking immunotherapies are now undergoing evaluation in clinical trials. The results of this study suggest potential benefits of CD47-blocking therapies in advanced stages of malignancy, such as peritoneal carcinomatosis.
